# Propofol Pretreatment Inhibits Liver Damage in Mice with Hepatic Ischemia/Reperfusion Injury and Protects Human Hepatocyte in Hypoxia/Reoxygenation

**DOI:** 10.5152/tjg.2023.21218

**Published:** 2023-11-01

**Authors:** Jing Li, Ruiqi Wang, He Chen, Yu Yang, Xinyuan Yang, Wei Wang

**Affiliations:** 1 Center for Translational Medicine, Xi’an Jiaotong University Medical College First Affiliated Hospital, Xi’an, Shaanxi, China; 2 Department of Obstetrics and Gynecology, Xi’an Jiaotong University Medical College First Affiliated Hospital, Xi’an, Shaanxi, China; 3 Department of Anesthesiology, Xi’an Jiaotong University Medical College First Affiliated Hospital, Xi’an, Shaanxi, China

**Keywords:** Propofol, hepatic ischemia/reperfusion injury, nuclear factor, erythroid-like 2, heme oxygenase 1

## Abstract

**Background/Aims:**

The major complication of liver resection is hepatic ischemia/reperfusion injury. Propofol appears to have organ-protective effects. Our study aimed to study the protective role of propofol against hepatic ischemia/reperfusion injury and the potential mechanisms.

**Materials and Methods:**

Mice and human hepatocytes (LO2) were used to establish 2 models: the ischemia/reperfusion injury model in vivo and the hypoxia/reoxygenation model in vitro, respectively. Alanine and aspartate aminotransferase serum levels were detected to evaluate the extent of hepatic cellular injury. Malondialdehyde, superoxide dismutase, glutathione, and catalase expression levels were measured to evaluate the oxidative damage in mice liver. Lactate dehydrogenase levels were detected for hepatocyte cytotoxicity severity. Nuclear factor, erythroid-like 2 and heme oxygenase 1 expression levels were detected.

**Results:**

In the ischemia/reperfusion model, propofol pretreatment significantly reduced the alanine aminotransferase and aspartate aminotransferase expression levels, alleviating the hepatic cellular injury. Propofol also protected the mice liver from oxidative damage. In the hypoxia/reoxygenation model, propofol pretreatment reduced lactate dehydrogenase expression levels, suggesting its protective effects in LO2 cells. Furthermore, propofol increased the nuclear factor, erythroid-like 2 and heme oxygenase 1 expression levels both in vivo and in vitro.

**Conclusion:**

Propofol acts through the nuclear factor, erythroid-like 2, and heme oxygenase 1 pathway to protect the mice liver against ischemia/reperfusion injury and hepatocytes against hypoxia/reoxygenation injury. Propofol should be used as an effective therapeutic drug for hepatic ischemia/reperfusion injury.

**Main Points**

- Propofol alleviated the hepatic ischemia/reperfusion injury in mice liver.
- Propofol protected mice liver from oxidative damage.
- Propofol had protective effects on hypoxia/reoxygenation hepatocyte.
- Propofol promoted nuclear factor, erythroid-like 2 (Nrf2) nuclear translocation, increasing Nrf2 and heme oxygenase 1 expression levels in mice liver and hepatocyte.

## Introduction

In recent years, the incidence of liver disease has continued to increase, showing a high demand for liver surgery than ever before.^1^ Studies demonstrated that nearly 360 million people worldwide are suffering from diseases caused by liver dysfunction.^2^ As blood flow will be temporarily blocked during the liver surgery, it causes injury to the liver tissue; such injury often fails to recover after the restoration of blood flow and the restoration of oxygen delivery to the previously ischemic tissues. Therefore, hepatic ischemia/reperfusion injury (HIRI) is caused, leading to a major complication during liver surgery.^3^ Hepatic ischemia/reperfusion injury is the leading cause of liver damage during liver transplantation and hepatectomy. Hepatic ischemia/reperfusion injury is also the main cause of graft dysfunction, leading to liver failure after transplantation.^4^ As a result, HIRI has become the topic of intense study worldwide attributed to its crucial effect.^5^

Propofol (2,6-diisopropylphenol) is a short-acting anesthetic drug that is often used in surgery.^6^ Previous studies have demonstrated that propofol exerts a strong antioxidative stress effect,^1^ and that the antioxidant properties of the drug can alleviate ischemia/reperfusion injury in the brain,^7^ heart muscle,^6^ and myocardial cells.^8^ Several mechanisms are involved in the process of HIRI.^9^ These mechanisms include oxygen-free radical increase, calcium overload, microcirculation dysfunction, and Kupffer cell activation. Among all these factors, the excessive generation of reactive oxygen species (ROS) is important.^10^ Studying the connection between HIRI and oxidative damage contributes to a deeper comprehending of the mechanisms underlying HIRI, thus helping in the design of new treatment strategies.

Nuclear factor, erythroid-like 2 (Nrf2), an antioxidant transcription factor, is critical in regulating cellular redox homeostasis as well as oxidative stress (OS) in the organs.^11^ In normal conditions, Nrf2 is localized in the cytoplasm and assembly with Keap1 (Kelch-like ECH-associated protein 1). When activated by exogenous or endogenous stimuli, Nrf2 falls off from Keap1, translocating into the nucleus and binding tightly with antioxidant-response elements, thereby regulating several relevant cytoprotective enzymes and antioxidant genes.^11^ Heme oxygenase 1 (HO-1), regulated by Nrf2, is a major phase II detoxifying stress-induced microsomal enzyme^12^ that protects the cells and organs from ROS-induced oxidative damage.^13^ Nuclear factor, erythroid-like 2 was reported to play a significant role in cerebral ischemia/reperfusion (I/R) injury^14^ and acute renal injury.^15^

We aimed to examine the effects of propofol in mice with I/R and in LO2 human liver cells grown under hypoxia/reoxygenation conditions. We also investigated the underlying mechanism of how propofol protects. Our findings suggested that propofol improved the liver function, possibly by activating Nrf2 nuclear translocation, which results in the upregulation of the downstream antioxidation factor HO-1 expression. This indicates that HO-1 plays an antioxidative role in HIRI. Since most studies investigate the effect of propofol post-treatment on ischemia/reperfusion injury, our study provides valuable information that propofol pretreatment inhibits liver damage in HIRI and provides another scientific evidence that propofol can be used as an effective therapeutic drug for HIRI.

## Materials and Methods

### Cell Culture and Hypoxia/Reoxygenation Model

LO2 cells were obtained from a normal human adult’s liver tissue. In this study, LO2 cells were obtained from the Shanghai Cell Bank (Shanghai, China). The RPMI-1640 medium with L-glutamine (RPMI) medium (Gibco, Carlsbad, California, USA) was used for cell culture. The concentration of fetal bovine serum was 10%. The cell experiment was divided into 3 groups. In the control group, LO2 cells were cultured at 37°C, 5% CO_2_ normally. In the hypoxia/reoxygenation (H/R) group, cells first underwent hypoxia for 15 hours, the hypoxia condition was 1% O_2_, and then cells were cultured for 6 hours under normal conditions. Propofol pretreatment plus Ischemia/Reperfusion Injury group, propofol (3.6 μg/mL) was added to the cells 24 hours before hypoxia for 15 hours and normal conditions for 6 hours (propofol or 2,6-diisopropylphenol: molecular weight: 178.27, Hwa, Xuzhou, China).

### Animals and Experimental Groups

The present study was approved by the Animal Ethics Committee of Xi’an Jiaotong University. Male C57 mice aged 6-8 weeks old (body weight 20-25 g) were purchased from W.T.L.H. Experimental Animal Technology (Beijing, China). Mice were raised under the following typical conditions: room temperature 25°C, relative humidity 50%, and 12 hours alternate day and night cycles. Mice were stochastically allocated to 3 groups: sham, hepatic I/R, and I/R + propofol group; each group contains 5 mice. In the group with sham operation, mice received the same surgery except for hepatic ischemia. In the I/R + propofol group, 2 hours before I/R surgery, mice received an intraperitoneal injection of propofol (50 mg/kg). Finally, the mice were euthanized after reperfusion for 4 h followed by obtaining blood and liver tissue samples.

### Hepatic Ischemia/Reperfusion Injury Model

The hepatic I/R injury model was established as follows: mice were anesthetized by pentobarbital sodium, the drug was injected intraperitoneally, and the dosage was 40 mg/kg. Then, a midline laparotomy was followed. The 70% hepatic ischemia model was established by blocking the blood supply to liver lobes. We use microaneurysm clamps to block the hepatic artery and portal vein, thus stopping the blood supply of the median and left lateral lobes for 45 minutes to establish ischemia. Reperfusion was started by moving the clamps away, and the reperfusion time was 4 hours. The abdominal incision was sutured using a 4-0 surgical thread. In the propofol pretreatment group, mice received an intraperitoneal injection of propofol (50 mg/kg) 2 hours before I/R surgery.

### Hematoxylin and Eosin Staining

The collected liver tissues were fixed in paraformaldehyde (pH 7.0, 10%) and embedded on paraffin. The tissues were then cut into 4 mm slices. The sections were stained with eosin solution after stained with hematoxylin. Photos were taken by Nikon Eclipse Ci (Tokyo, Kanto, Japan).

### Immunohistochemistry

To detect the Nrf2 expression level in mice liver, immunohistochemistry (IHC) was performed. Briefly, the tissue sections were incubated with the Nrf2 antibody overnight (the hybridization temperature was 4°C) (Proteintech, 110 kDa, 16396-1-AP) and HO-1 antibody (Proteintech, 33 kDa, 10701-1-AP) at a dilution of 1 : 200. Photos were taken by Nikon Eclipse Ci.

### Assay for Serum Biochemical Values

Blood samples were centrifuged at 4°C, the centrifugal speed was 1500 *g* per minute, and the centrifugal time was 15 minutes. Serum alanine/aspartate aminotransferase (ALT/AST) expression levels were detected using a biochemical analyzer (Nanjing, Jiangsu Province, China).

### Assay for Malondialdehyde, Superoxide Dismutase), Catalase, and Glutathione in the Liver Homogenate

Mouse liver tissue homogenates were prepared and centrifuged. The oxidative damage was detected by malondialdehyde (MDA), the antioxidant activity was measured by 2 antioxidant enzymes superoxide dismutase (SOD) and catalase (CAT) and the antioxidant glutathione (GSH). All the assay kits were bought from Nanjing Jiancheng.

### Cytotoxicity Assay

Lactate dehydrogenase (LDH) cytotoxicity assay kit (C0016, Beyotime, Shanghai, China) was used to assess cell cytotoxicity. The supernatant was first added into a 96-well plate and then mingled with the reaction mixture before incubating at 37°C for half an hour; finally, the 490-nm absorbance was detected with a biochemical analyzer (BioTek, Winooski, Vt, USA) to determine the LDH activity.

### Immunofluorescence Staining

LO2 cells were first washed with phosphate-buffered saline (PBS) followed by 4% Paraformaldehyde (PFA) fixed for 10 minutes. Then, 0.1% Triton X-100 was used to penetrate the cell membrane, and the penetration time was 10 minutes. Next, the antigen was blocked by 5% bovine serum albumin for 1 hour incubation at room temperature. Then, the anti-Nrf2 antibody was added and incubated at 4°C overnight. Finally, the slides were hybridized with a fluorescent-labeled secondary antibody, the dilution ratio of the antibody was 1:50, and the hybridization condition was 1 hour at 25°C. Slips were mounted with a mounting medium after washing with PBS 3 times and then observed through a confocal fluorescent microscope.

### Quantitative Reverse Transcriptase Polymerase Chain Reaction

Total mRNA was extracted by TRIzol reagent (Invitrogen [Carlsbad, California, USA]). cDNA was synthesized by cDNA Synthesis Kit (Thermo [Stony Creek, California, USA], catalog number: #K1622). The target gene expression levels were measured with the TB Green Premix (TAKARA [Osaka city, Osaka prefecture, Japan], catalog number: RR820A) using CFX 96 (Bio-Rad [Hercules, California,USA]). The primer sequences of the related genes were as follows:

Nrf2: 5’-GAGAGCCCAGTCTTCATTGC-3’ and 5’-TTGGCTTCTGGACTTGGAAC-3’.

HO-1:5’-TCCTGGCTCAGCCTCAAATG-3’ and 5’-CGTTAAACACCTCCCTCCCC-3’.

β-Actin: 5’-AGCACTGTGTTGGCGTACAG-3’ and 5’-TCCCTGGAGAAGAGCTACGA-3’,

### Protein Extraction and Western Blotting

Nucleoproteins were extracted from the mouse liver tissues or hepatocyte by using a nuclear extraction kit (Abcam [Cambridge, London, United Kingdom], ab113474). Total proteins from liver tissues or hepatocytes were extracted using a Radio Immunoprecipitation Assay (RIPA) lysis buffer. The proteins were centrifuged for 30 minutes at a speed of 12 000 rpm for collecting the supernatant, and the centrifugation is required at 4°C. The extracted proteins were quantified using a bicinchoninic acid assay (BCA) kit (Beyotime). Next, they were denatured using a 5× loading buffer at 95°C for 5 minutes, and the denatured proteins were separated by sodium dodecyl sulphate-polyacrylamide gel electrophoresis (SDS-PAGE). Then, proteins were transferred to the nitrocellulose filter membrane (NC) before blocking with 5% milk. After blocking, the primary antibodies were added to the membranes for hybridization overnight, and the reaction temperature was 4°C. The antibodies used were Nrf2 (Proteintech, 16396-1-AP, 110 kDa, 1:1000), HO-1 (Proteintech, 10701-1-AP, 33 kDa), and β-actin (Proteintech, 60008-1-lg, 42 kDa). The membranes were hybridized with secondary antibodies for 1 hour at room temperature; finally, the proteins were measured with the chemiluminescence detection system (GE [Boston, Massachusetts, USA]).

### Statistical Analysis

Data were analyzed by the GraphPad Prism 9 software. Biochemical assays were repeated 3 times. Data are expressed as means ± SEM. Significance was analyzed by 1-way ANOVA with post hoc contrasts by Student–Newman–Keuls test. *P* < .05 was considered statistically significant.

## Results

### Propofol Alleviates Hepatic Ischemia/Reperfusion Injury in the Mouse Liver

**Figure 1.**
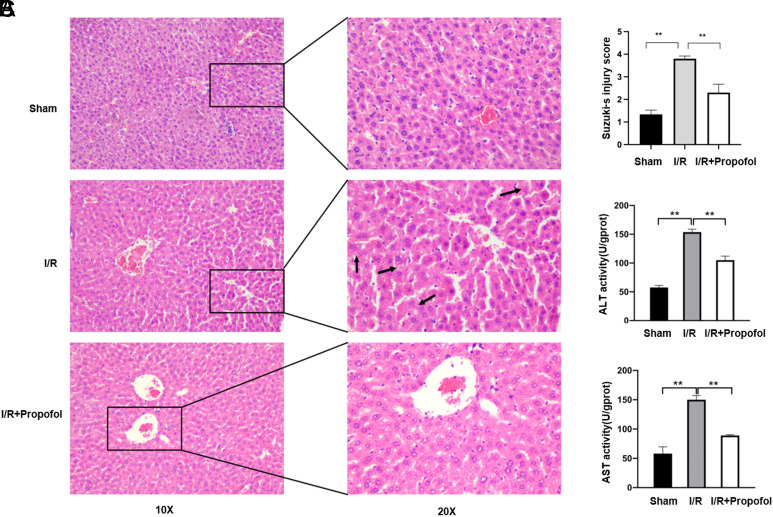
Hepatic protection by propofol versus hepatic ischemia/reperfusion injury in vivo. (A) Histopathological changes in the liver tissues. Hematoxylin and eosin (HE)-stained liver tissue samples. Black arrow indicates vacuolar degeneration. In the ischemia–reperfusion (I/R) group, more vacuolar degeneration and hepatocyte swelling were detected, whereas fewer alterations in the structure were detected in the I/R + propofol group. (B) Suzuki’s injury score of HE. (C) Hepatic cellular injury as evaluated by the serum alanine aminotransferase (ALT) and aspartate aminotransferase (AST) levels. *P < .05, **P < .01.

The hematoxylin and eosin (HE) images of the liver tissues and Suzuki’s injury scores are presented in Figure 1A and 1B. The liver tissue architecture was normal in the sham group, but it demonstrated typical hallmarks of severe injuries, such as hepatocyte degeneration and swelling, hepatic necrosis, and inflammatory cell infiltration, in the I/R group. In contrast, there was relative preservation of the tissue structure in the I/R + propofol group, indicating an apparent reduction in the damage caused to liver tissues than the I/R group. Blood and histological samples were examined to determine the extent of hepatocellular damage. The ALT and AST serum levels were distinctly grown in the I/R group than in sham. In contrast, in the I/R + propofol group, ALT and AST serum levels were evidently declined (*P* < .01) (Figure 1C).

### Propofol Increases the Antioxidant Capacity of the Mouse Liver

**Figure 2.**
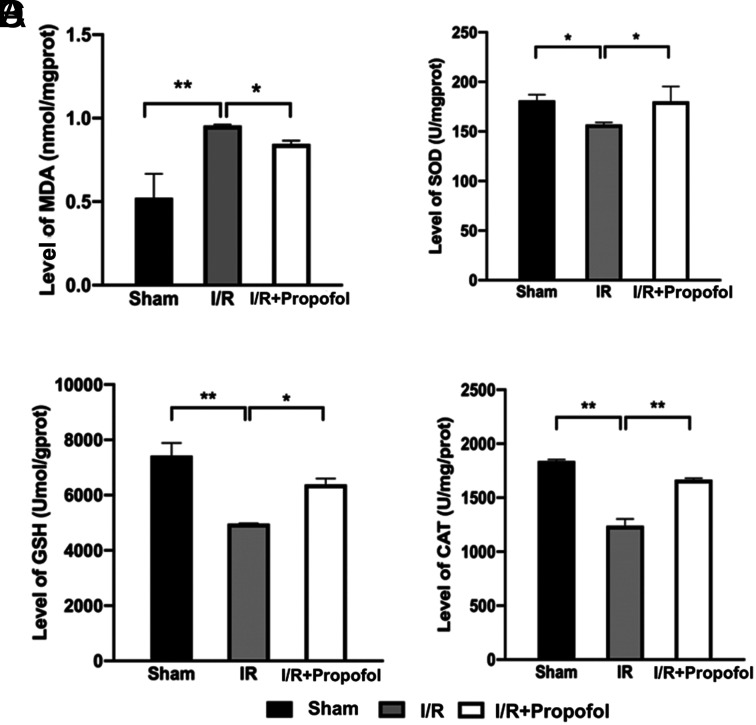
Propofol increased the ability of the mouse liver to resist oxidative damage. (A) Alterations of malondialdehyde (MDA). (B) Alterations of superoxide dismutase (SOD). (C) Alterations of glutathione (GSH). (D) Alterations of catalase (CAT). *P < .05, **P < .01.

Oxidative damage is inevitable during a hepatic I/R injury; therefore, we analyzed 1 oxidative activity marker MDA and 3 antioxidative activity markers, such as antioxidant enzymes SOD and CAT and an antioxidant GSH, in the mouse liver homogenate. Malondialdehyde, which reflects lipid peroxidation and cellular damage, was increased sharply in the I/R group than sham but was declined in the I/R + propofol group than the I/R group (Figure 2A). In contrast with the sham group, the expression levels of the antioxidants SOD, CAT, and GSH were reduced in the I/R group (*P* < .05), while in the I/R + propofol group, their expression levels were restored (*P* < .05) (Figure 2B–D). These findings implied that propofol reduced the I/R-induced oxidative damage to the mouse liver.

### Propofol Promotes Nuclear Factor, Erythroid-Like 2 Translocation Into the Nucleus and Upregulates Heme Oxygenase 1 in the Mouse Liver

**Figure 3.**
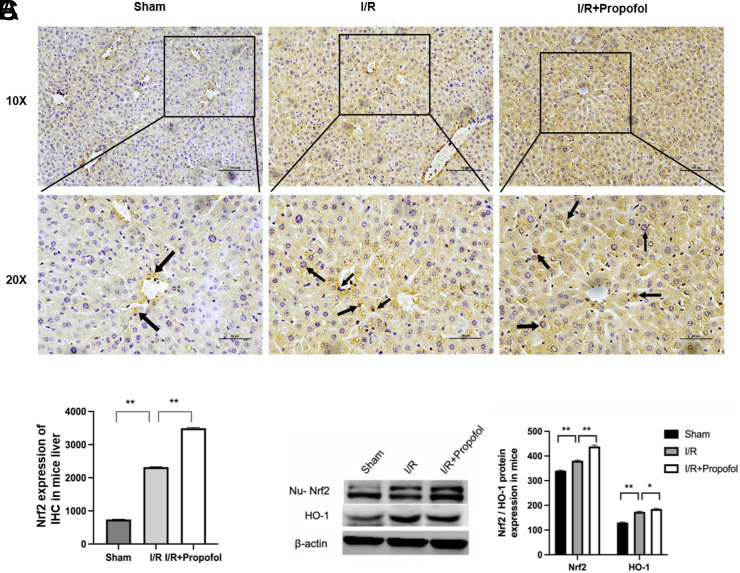
Propofol enhanced the nuclear factor, erythroid-like 2 (Nrf2) and heme oxygenase 1 (HO-1) protein expression levels in the mouse liver. (A) Nrf2 expression as detected by immunohistochemical analysis in each group. Black arrows indicate the location of Nrf2. (B) Histogram of the Nrf2 expression of immunohistochemistry in the mouse liver. (C) The Nu-Nrf2 and HO-1 protein expression of the mouse liver and the histogram of Nu-Nrf2 and HO-1 in western blotting. *P < .05, **P < .01.

To elucidate the underlying mechanisms of propofol’s protective role, Nrf2 expression and HO-1 expression were detected in the mouse liver. Immunohistochemical results revealed that the Nrf2 expression level was enhanced not only in the I/R group but also in sham and I/R + propofol group; furthermore, the staining in the I/R + propofol group was deeper (Figure 3A). The histogram of the Nrf2 expression of IHC in the mouse liver is presented in Figure 3B. Furthermore, we detected the alteration of the Nrf2 nucleoprotein (Nu-Nrf2) expression level between different groups, and Nu-Nrf2 expression levels were raised both in the I/R and in the I/R + propofol groups. Additionally, a comparison of the I/R group and I/R + propofol group suggested that the Nu-Nrf2 expression level was higher in the latter group. The HO-1 expression level was increased in the I/R and I/R + propofol groups than sham (Figure 3C).

### Propofol Has Protective Effects on Hypoxia/Reoxygenation Cells

**Figure 4.**
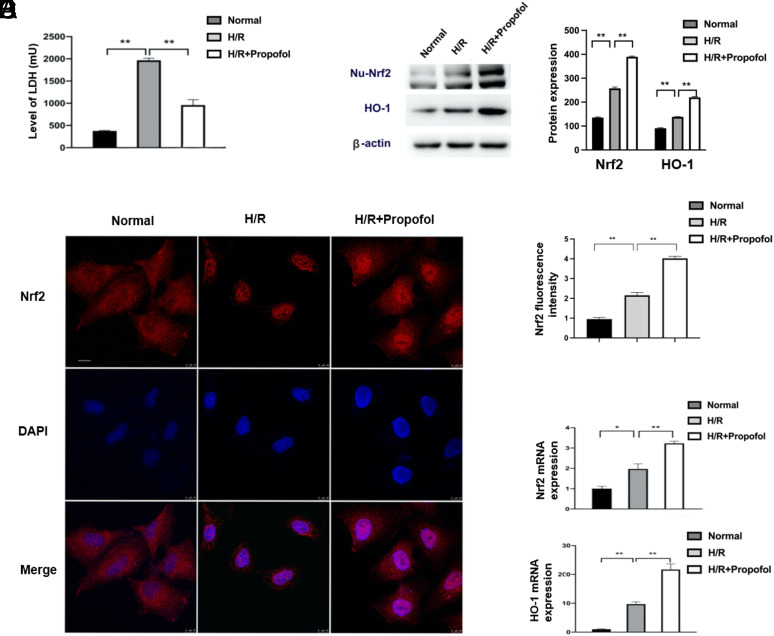
Propofol exhibited protective effects in hepatocytes in the hypoxia/reoxygenation cell model. (A) Lactate dehydrogenase (LDH) expression levels in each group. (B) The nuclear factor, erythroid-like 2 (Nrf2) nucleoprotein (Nu-Nrf2) protein expression level and heme oxygenase 1 (HO-1) protein expression level. (C) The Nrf2 expression in each group. Scale bar, 10 μm. (D) Histogram of Nrf2 fluorescence intensity in LO2 cells. (E) The Nrf2 mRNA expression level and HO-1 mRNA expression level. *P < 0.05, ** *P *< .01.

To verify the protective role of propofol in HIRI, an H/R model was set up in human hepatocytes. Lactate dehydrogenase is a sensitive marker of cellular damage. When the cell membrane is lysed, LDH is released from the cytoplasm to the cell culture. Therefore, LDH is widely used in evaluating cytotoxicity. In our study, LDH expression was raised distinctly in the H/R group in contrast with sham (*P *< .01), implying the successful establishment of the H/R model (Figure 4A). The LDH was obviously reduced (*P *< .01) in the H/R + propofol group than the H/R, suggesting that propofol can protect LO2 cells from cytotoxicity (Figure 4A). Immunofluorescence results revealed that the Nrf2 nucleoprotein expression enhanced evidently in the H/R group than sham and that the Nrf2 protein expression was increased both in the cytoplasm and in the nucleus in the H/R + propofol group than H/R (Figure 4C and 4D). Nuclear factor, erythroid-like 2 and HO-1 mRNA expression levels and Nrf2 nucleoprotein and HO-1 protein expression levels were raised simultaneously in the H/R group than sham (*P *< .05). Meanwhile, Nrf2 and HO-1 expression levels were all raised in the H/R + propofol group than the H/R (*P *< .01) (Figure 4B and 4E). All the in vitro results were in line with in vivo results.

## Discussion

In the present study, propofol pretreatment alleviated HIRI in mice and ameliorated H/R damage in human hepatocytes (LO2). It also reduced the damage caused by I/R in the liver and H/R in hepatocytes and increased the Nrf2 expression and HO-1 expression.

Nuclear factor, erythroid-like 2 is essential to redox homeostasis. It is the key transcription factor regulating antioxidant defense systems in response to OS.^15^ Nuclear factor, erythroid-like 2 equips cells with a complex endogenous protection system to fight against the OS and Nrf2 regulates the antioxidant capacity of cells through binding to the antioxidant response element of the target genes.^16^ The antioxidant capacity of the Nrf2 system is regulated by the acetylation–deacetylation state of Nrf2. After being deacetylated by Sirt1, the expression of Nrf2 and the downstream antioxidant genes was enhanced, leading to the improvement of antioxidant capacity.^17^ Nuclear factor, erythroid-like 2 can activate a variety of cytoprotective genes such as HO-1 and NAD(P)H quinone oxidoreductase-1.^18^ The Nrf2/HO-1 signaling pathway functions as the primary cellular sensor for OS.^18^ The Nrf2/HO-1 pathway attenuates oxidative damage, since its activation is associated with antioxidant proteins such as SOD1.^19^ Heme oxygenase 1 is a powerful antioxidant^20^ and plays an important role in maintaining the redox balance of cells.^18^

Ischemia/reperfusion injury affects the liver function after liver transplantation or resection. Several studies have suggested that OS is the main reason in hepatocytes suffering H/R injury.^21^ The body generates reactive oxygen through aerobic metabolism, and the balance between the generation of ROS and the elimination of ROS was broken. Excessive accumulation or the generation of free radicals due to an imbalance in the antioxidative system is harmful. In ischemia, the activation of xanthine oxidase leads to excessive generation of hypoxanthine and single electrons. In reperfusion, the molecular oxygen combines with the single electron to produce free radicals of oxygen and lipid, damaging the cell membrane, mitochondrial membrane, and lysosome. The damaged lysosomes release numerous enzymes, leading to a series of pathologic alterations.^22^

To date, several studies have focused on how propofol protects I/R injury in the brain,^10^ intestine,^23^ and lungs.^24^ Some of these studies indicated that propofol ameliorates oxygen utilization in tissues, thus regulating oxygen metabolism.^25^ Therefore, propofol possibly alleviates hypoxia caused by disordered energy metabolism in the tissues.^26^

In our study, in mice with hepatic I/R injury, ALT and AST serum levels were increased significantly, but their levels reduced after propofol pretreatment. Histopathological analysis indicated that propofol significantly decreased hepatocyte necrosis and swelling, inflammatory cell infiltration, and hepatocyte degeneration. These findings support the fact that propofol protects liver against I/R injury. The 4 OS markers were measured to evaluate the oxidation levels of the liver. We found that the MDA expression level was notably reduced, while SOD, GSH, and CAT expression levels increased in the I/R + propofol group. Further, we found that Nrf2 nucleoprotein expression levels were raised in the I/R and I/R + propofol groups than sham. The Nu-Nrf2 expression levels were also obviously raised in the I/R + propofol group compared with the I/R group. Moreover, HO-1 expression levels were increased in the I/R + propofol group than sham and I/R. These findings suggest that propofol induced Nrf2 transferred into nucleus regulating HO-1 expression and enhancing its antioxidant capacity. Recent evidence suggests that Nrf2 is localized in the cytoplasm, Nrf2 and is the substrate protein of protein kinase C. Following activation, Nrf2 moves into the nucleus, regulating antioxidant enzymes, such as HO-1. During I/R injury, red blood cells are damaged, and there is increased resistance to blood flow. The heme released from the red blood cells accelerates oxidation process. Heme oxygenase 1 is a rate-limiting enzyme that catalyzes heme into free iron, carbon monoxide, and biliverdin (BV).^27^ Carbon monoxide and BV primarily mediate the cytoprotective effects of HO-1 through anti-apoptosis, anti-inflammation, and vasodilatation.^28^ Further, the molecular oxygen is consumed during heme degradation, thereby reducing ROS production and exerting its protective effects during I/R injury.

The drawback of our study was the small number of animals, although it meets the statistical requirement, but the results of the small sample group can only stand for this specific research group; therefore, more research especially in patient samples is required to confirm the results in larger groups.

**Figure 5.**
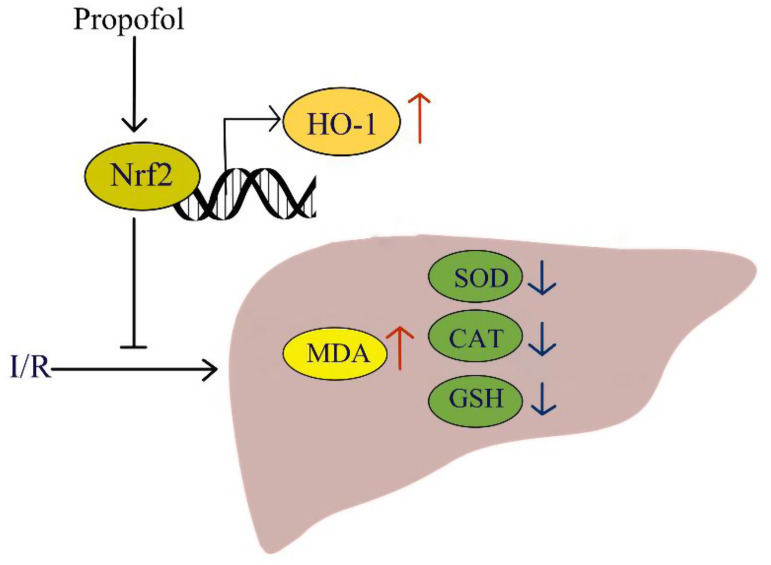
Schematic illustration of the potential mechanisms of propofol protects against hepatic ischemia/reperfusion injury. CAT, catalase; I/R, ischemia/reperfusion; GSH, glutathione; HO-1, heme oxygenase 1; MDA, malondialdehyde; Nrf2, nuclear factor, erythroid-like 2; SOD, superoxide dismutase.

Briefly, propofol significantly ameliorated HIRI and improved liver function by reducing the oxidative damage and enhancing the liver’s antioxidant capacity. Besides, it promoted Nrf2 translocation into the cell nucleus and subsequently upregulated the HO-1 expression in the mouse liver as well as human hepatocytes. Overall, our study demonstrated that propofol protects the tissues from HIRI by enhancing the Nrf2/HO-1 pathway (Figure 5). This study may give insights to develop new therapeutic strategies for HIRI.
